# Nigral glucose metabolism as a diagnostic marker of neurodegenerative parkinsonian syndromes

**DOI:** 10.1038/s41531-022-00392-x

**Published:** 2022-09-29

**Authors:** Nils Schröter, Ganna Blazhenets, Lars Frings, Wolfgang H. Jost, Cornelius Weiller, Michel Rijntjes, Philipp T. Meyer, Joachim Brumberg

**Affiliations:** 1 Department of Neurology, Medical Center – University of Freiburg, Faculty of Medicine, University of Freiburg, Freiburg, Germany; 2 Department of Nuclear Medicine, Medical Center – University of Freiburg, Faculty of Medicine, University of Freiburg, Freiburg, Germany; 3 Center for Geriatrics and Gerontology Freiburg, Medical Center - University of Freiburg, Faculty of Medicine, University of Freiburg, Freiburg, Germany; 4 Parkinson-Klinik Ortenau, Wolfach, Germany

**Keywords:** Diagnostic markers, Parkinson's disease, Diagnostic markers

## Abstract

Parkinson’s disease (PD), multiple system atrophy (MSA), and progressive supranuclear palsy (PSP) are characterized by nigrostriatal degeneration. We used [^18^F]FDG PET to assess glucose metabolism of the substantia nigra (SN) in patients with these diseases and evaluated its ability to discriminate neurodegenerative parkinsonian syndromes (NP) from controls. We retrospectively evaluated [^18^F]FDG PET scans of 171 patients with NP (*n* = 115 PD, *n* = 35 MSA, *n* = 21 PSP) and 48 controls (13 healthy controls [HC] and 35 control patients). Mean normalized bilateral [^18^F]FDG uptake in the SN was calculated and compared between groups with covariance and receiver operating characteristic (ROC) analyses (selection of the optimal cut-off required a minimum specificity of 90% to meet the clinical need of a confirmatory test). PD patients were additionally stratified by the expression of the well-established PD-related metabolic pattern (PDRP; elevated expression defined as 2 standard deviations above the mean value of HC). [^18^F]FDG uptake was significantly lower in NP (Cohen’s *d* = 1.09, *p* < 0.001) and its subgroups (PD, *d* = 1.10, *p* < 0.001; MSA, *d* = 0.97, *p* < 0.001; PSP, *d* = 1.79, *p* < 0.001) than in controls. ROC analysis for discriminating NP vs. controls revealed an area under the curve of 0.81 and a sensitivity and specificity of 56 and 92%. Moreover, nigral metabolism was below the cut-off in 60% of PD patients without elevated PDRP expression. Glucose metabolism of the SN can distinguish patients with NP from controls with good diagnostic accuracy and can be used as a marker of nigral degeneration. Its evaluation is particularly valuable in PD patients without elevated PDRP expression and may thus help to narrow the diagnostic gap of [^18^F]FDG PET in neurodegenerative parkinsonism (i.e., identification of patients with PD without cortical involvement).

## Introduction

Nigrostriatal degeneration is the histopathological hallmark of neurodegenerative parkinsonian syndromes (NP) and can be detected by means of presynaptic dopaminergic neuroimaging^1^. Current guidelines account for this by recommending dopaminergic imaging for the clinical work-up of parkinsonian syndromes, but they do not support its use to distinguish between different neurodegenerative parkinsonian syndromes^2–4^. The evaluation of cerebral glucose metabolism with [^18^F]fluorodeoxyglucose (FDG) positron emission tomography (PET) instead allows to differentiate between Parkinson’s disease (PD), multiple system atrophy (MSA), and progressive supranuclear palsy (PSP) with high accuracy^5^. Patients with PD with cortical involvement (observed in up to 50% of a typical clinical population^6,7^), especially those with mild cognitive impairment [PD-MCI] and PD dementia [PDD]), MSA, and PSP can often be easily diagnosed by means of typical (sub-)cortical metabolic patterns. In turn, patients with PD without cortical involvement may present with only subtle metabolic abnormalities like relative striatal hypermetabolism. These may be hardly distinguishable from normality and constitute a clinical challenge^5,6^. In those cases, the differential diagnosis with [^18^F]FDG PET relies on the exclusion of other forms of NP and should be supported by the proof of nigrostriatal degeneration (e.g., with dopamine transporter [DAT] single-photon emission computed tomography [SPECT]).

Recent findings in PD patients revealed that metabolism in the substantia nigra pars compacta is reduced and correlates with putaminal dopamine synthesis capacity^8^. This suggests that nigral glucose metabolism may be a suitable measure for nigral degeneration. The aim of this study was, therefore, to examine the diagnostic accuracy of nigral metabolism measured with [^18^F]FDG PET in delineating NP from controls (healthy controls [HC] and control patients). Furthermore, we assessed the diagnostic value of reduced metabolism in the substantia nigra in a subgroup of PD patients who did not show an elevated expression of the well-characterized PD-related metabolic pattern (PDRP)^9^).

## Results

### Patients

There were no group differences in terms of sex and age at PET imaging between patients with NP, PD, or MSA and controls (all *p* > 0.1). However, patients with PSP were older than controls (*p* < 0.001), MSA and PD (both *p* < 0.01) and prevalence of male patients was lower in MSA compared to PD (*p* < 0.05). As expected, we observed significant differences in disease duration between groups: PD patients had the longest disease duration, and PSP patients had the shortest disease duration (ANOVA, group factor: *p* < 0.001, *F* = 12.6, degrees of freedom (df) = 3), for details, see Table 1.

**Table 1:** Demographic and clinical characteristics of patient groups.

	PD	MSA	PSP	NP	Controls
n	115	35	21	171	48
Sex, male %	63	37	52	56	52
Age, years mean (SD)	66.7 (8.9)^b^	64.3 (9.1)^c^	73.9 (7.8)^a,b,c^	67.1 (9.2)	66.2 (8.5)^a^
Disease duration, years mean (SD)	9.1 (5.7)^d,e^	4.4 (3.7)^d^	3.0 (1.6)^e^	7.4 (5.5)	NA
Hoehn & Yahr mean (SD)	2.89 (0.76)	3.20 (0.72)	3.14 (0.36)	2.99 (0.72)	NA
UPDRS III (off) mean (SD)	44.86 (18.64)	47.91 (14.15)	42.53 (10.07)	45.27 (17.13)	NA
Nigral [^18^F]FDG uptake mean (SD)	0.80 (0.06)^**^	0.81 (0.08)^*^	0.77 (0.06)^**^	0.80 (0.07)^**^	0.87 (0.05)

Hoehn & Yahr stage at PET imaging was available for all patients except three patients with MSA. UPDRS III (off) score was available for *n* = 111 PD, *n* = 33 MSA, and *n* = 15 PSP patients.

*PD* Parkinson’s disease, *MSA* multiple system atrophy, *PSP* progressive supranuclear palsy, *NP* neurodegenerative parkinsonian syndromes, *n* number of subjects, *SD* standard deviation, *UPDRS III* (*off*) unified Parkinson’s disease rating scale motor part 3 (off medication).

Differences in comparison to controls: *p < 0.001, **p < 0.0001.

^a–e^Significance of pairwise comparisons: a, b, c *p* < 0.01; d, e *p* < 0.001.

### Imaging

**Fig. 1:**
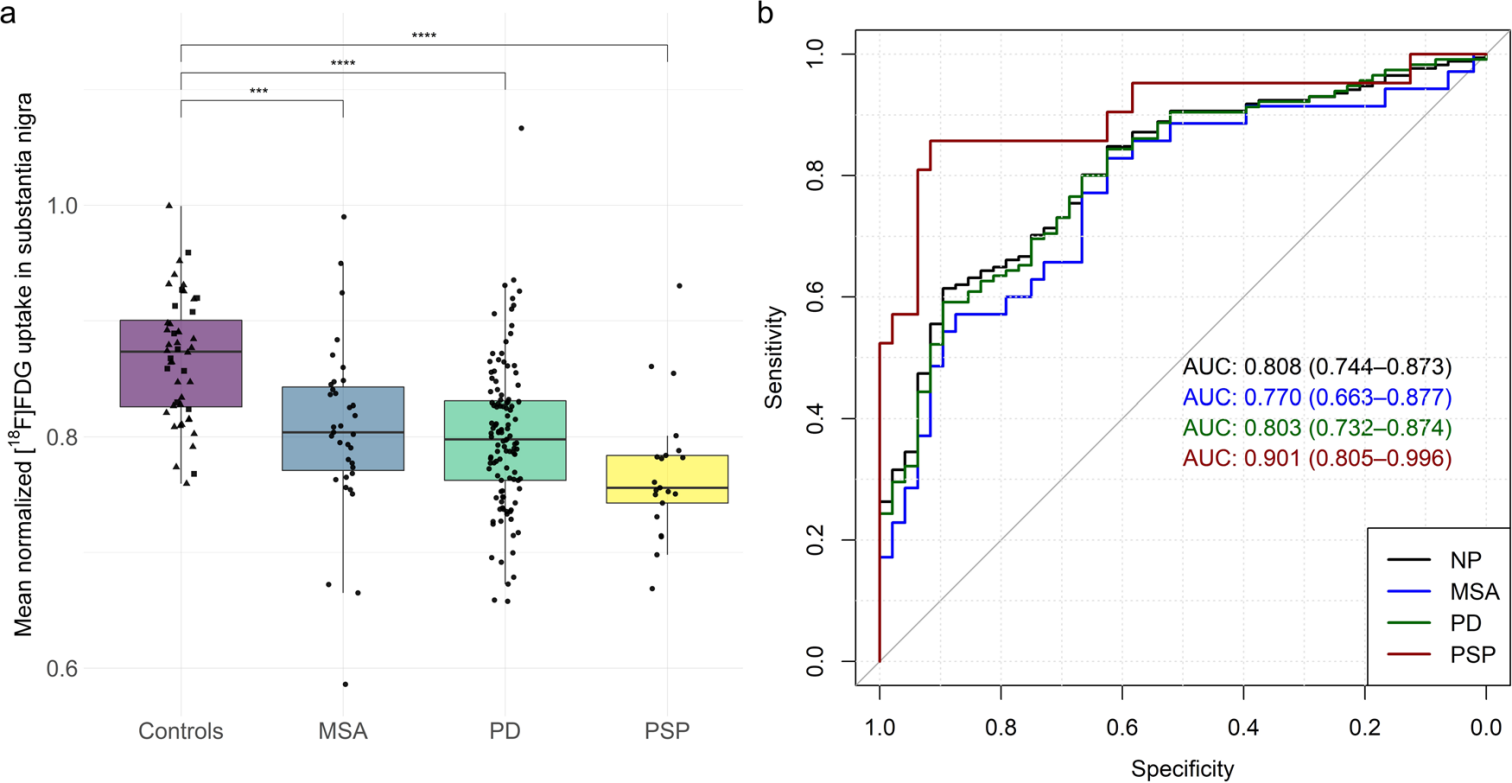
Nigral glucose metabolism in patients with neurodegenerative parkinsonism. **a** Boxplots of mean normalized [^18^F]FDG uptake of the substantia nigra across diagnostic groups and controls. Squares and triangles indicate healthy controls and control patients, respectively. Dots represent individual patients’ values. Centerline, median; box limits, upper and lower quartiles; whiskers, 1.5x interquartile range; points, outliers. Significance threshold: ****p < 0.0001, ***p < 0.001. **b** Receiver operating characteristics curve (ROC) for each diagnostic group. Areas under the ROC curves (AUC) and their 95%-confidence intervals (in parenthesis) are given. MSA multiple system atrophy, PD Parkinson’s disease, PSP progressive supranuclear palsy, NP neurodegenerative parkinsonian syndromes.

Mean normalized [^18^F]FDG uptake was similar in healthy controls (mean ± SD: 0.88 ± 0.05) and control patients (0.86 ± 0.05), that is why we performed all subsequent analyses with one combined control group only. [^18^F]FDG uptake was significantly different among patient groups and controls (ANCOVA, factor group: *p* < 0.001, *F* = 17.5, df = 3). Post hoc analyses indicated that nigral uptake was significantly lower in NP (*d* = 1.09, *p* < 0.001) and its subgroups (PD, *d* = 10; MSA, *d* = 0.97; PSP, *d* = 1.79; all *p* < 0.001) compared to controls (Fig. 1a). This was also confirmed in the sub-cohort with available [^123^I]FP-CIT SPECT (NP, *d* = 1.11, *p* < 0.001; PD, *d* = 1.06, *p* < 0.001; MSA, *d* = 1.61, *p* > 0.1; PSP, *d* = 2.57, *p* < 0.001; Supplemental Fig. 1a). ROC analysis indicated that a cut-off of 0.80 for normalized [^18^F]FDG uptake in the substantia nigra discriminated between patients and controls with 92% specificity and 56% sensitivity (ROC AUC = 0.81). For subgroups, ROC analyses showed discrimination between patients and controls with a ROC AUC of 0.80, 0.77, and 0.90 for PD, MSA, and PSP, respectively (Fig. 1b).

**Fig. 2:**
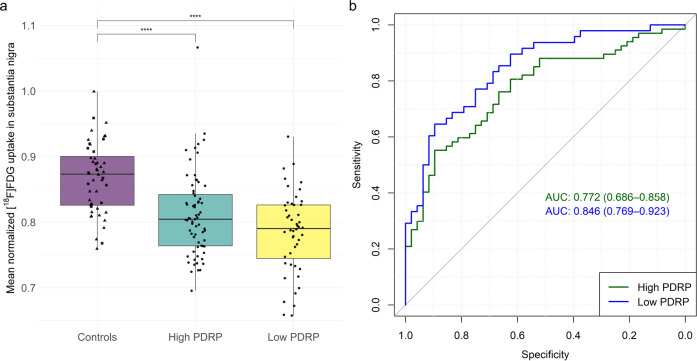
Nigral glucose metabolism in patients with Parkinson’s disease. **a** Boxplots of mean normalized [^18^F]FDG uptake of the substantia nigra in patients with high and low Parkinson’s disease-related pattern (PDRP) expression in comparison to controls. Squares and triangles indicate healthy controls and control patients, respectively. Dots represent individual patients’ values. Centerline, median; box limits, upper and lower quartiles; whiskers, 1.5x interquartile range; points, outliers. Significance threshold: ****p < 0.0001. **b** Receiver operating characteristics curve (ROC) for both groups. Areas under the ROC curves (AUC) and their 95%-confidence intervals (in parenthesis) are given. PD Parkinson’s disease.

Elevated expression of the PDRP (i.e., >13.78; mean ± SD in HC = 3.86 ± 4.96) was detected in 67 (58%) PD patients (“high PDRP score”; 25.50 ± 9.82). In those PD patients without elevated PDRP expression (*n* = 48, “low PDRP score”; 4.85 ± 5.61), the aforementioned cut-off of normalized [^18^F]FDG uptake of the substantia nigra (0.80) allowed for separation from controls with 60% sensitivity and 92% specificity (ROC AUC = 0.85; Fig. 2b). Thus, a total of 29 PD cases (60%) with a PDRP score within the normal range were correctly identified as suffering from PD, among them 18 with available [^123^I]FP-CIT SPECT confirming nigrostriatal degeneration. Reduced [^18^F]FDG uptake in the substantia nigra was present in patients with high PDRP score (*d* = 0.94, *p* < 0.001), low PDRP score (*d* = 1.43, *p* < 0.001), early (*d* = 1.17, *p* < 0.001) and late (*d* = 1.10, *p* < 0.001) PD stages (Supplemental Fig. 1b), and in PD patients with a disease duration <5 years (*d* = 1.14, *p* < 0.001 and >5 years (*d* = 1.12, *p* < 0.001).

### Correlation with clinical parameters

At the time point of PET imaging, UPDRS motor part 3 scores in medication-off condition were available in 97% of PD patients (*n* = 111). Forty-five of these patients underwent [^123^I]FP-CIT SPECT within 3 months of PET imaging. No significant association was found between mean normalized [^18^F]FDG uptake of the substantia nigra and disease duration or motor scores, whereas [^123^I]FP-CIT specific binding potential in putamen showed a moderate negative correlation with UPDRS scores (*r* = −0.45, *p* = 0.002).

## Discussion

This study demonstrates that the evaluation of glucose metabolism in the substantia nigra with [^18^F]FDG and a fully digital PET/CT system can differentiate NP and its subgroups PD, MSA, and PSP from healthy controls and control patients with high diagnostic accuracy. Moreover, it is particularly useful in PD patients without a typical metabolic pattern of cortical involvement. Thereby, [^18^F]FDG PET provides additional and valuable information in the work-up of patients with suspected NP.

DAT SPECT is a well-established imaging standard for identifying NP by means of striatal DAT loss (sensitivity of 78–97% at a specificity >95%)^10^ leading to changes in diagnosis and management^11^. In comparison to SPECT, the assessment of nigral metabolism with [^18^F]FDG PET shows lower sensitivity of 56%, while the specificity of 92% is almost comparable. However, together with the high diagnostic value of (sub-)cortical metabolic patterns^5^, the combined assessment of nigral and cerebral metabolism enables both evaluation of the nigrostriatal system and differential diagnosis of NP. The clinical benefit of confirming pathological DAT availability is negligible in patients with a clear metabolic pattern of PD (i.e. hypometabolism in the temporoparietooccipital cortex), MSA (i.e., hypometabolism in the posterior putamen and cerebellum), or PSP (i.e., hypometabolism in the medial and dorsolateral frontal cortex, caudate, and thalamus)^5^. However, in the subgroup of PD patients without elevated PDRP expression, dopaminergic imaging substantially increases diagnostic confidence. Thus, the present study suggests that evaluating nigral metabolism may supersede dopaminergic imaging in up to 60% of patients without elevated PDRP expression and thereby considerably improve the diagnostic value of [^18^F]FDG PET in the differential diagnosis of NP. Overall, 95 out of 115 PD patients (83%) showed metabolic abnormalities detectable with [^18^F]FDG PET (cortical involvement and/or hypometabolism of the substantia nigra) in the present study. Consequently, a diagnostic algorithm that favors [^18^F]FDG PET over DAT imaging for the initial evaluation of clinically uncertain parkinsonian syndromes may reduce the number of scans, associated costs, and burden to the patient. Further prospective studies are needed to define optimal clinical algorithms, confirm the present findings also at disease onset, and assess the clinical impact of the evaluation of nigral metabolism.

Reduced metabolism of the substantia nigra was observed in each subgroup of NP with comparable reductions in patients with PD (mean normalized [^18^F]FDG uptake: 0.80) and MSA (0.81), while the reduction was slightly more pronounced in patients with PSP (0.77). This is consistent with neuropathological findings of nigral cell loss in these three diagnostic entities^12–15^. However, the variance of nigral metabolism in each group and the overlap with the control cohort is noticeable and might be partially related to the clinical features of included subjects. Although we did not observe an association between nigral metabolism and disease duration or motor scores in the PD group, heterogeneity in terms of disease stages within each group may cause variable severity of brainstem involvement^16^. Furthermore, disease variants, like MSA with predominant parkinsonism or MSA with cerebellar ataxia and PSP Richardson syndrome, PSP with predominant parkinsonism or PSP with frontal presentation exhibit varying degrees of brainstem pathology and nigral cell loss^12,15,17^. This may, to a certain degree, explain differences in the level of glucose metabolism observed in our PSP cohort, in which PSP with predominant frontal presentation (*n* = 3) and corticobasal syndrome (*n* = 3) showed a less pronounced decrease compared to PSP Richardson syndrome (data not shown). However, due to the small sample sizes of the subgroups, we cannot draw firm conclusions and more data are needed, particularly of patients early during the disease course, to elucidate when metabolic changes in the substantia nigra start to occur.

Methodological limitations of PET data analysis must be considered: cortical involvement occurs in all diseases and advances with disease progression. For instance, it is associated with reduced [^18^F]FDG uptake in posterior temporoparietal and occipital areas in PD, especially in PD-MCI and PDD^5,18^. We accounted for this by normalizing PET data to the mean uptake of brain parenchyma after the exclusion of regions with typical MSA-, PSP-, and PD-related hypometabolism. However, decreased cortical uptake may also be present in other regions or may occur more globally with disease progression and therefore balance and even outweigh uptake reductions in the substantia nigra. This may well explain why we did not observe correlations with clinical parameters or relevant differences in substantia nigra metabolism of PD patients with early and late disease stages. The choice of white matter (for all groups) and the pons (for PD and controls) as alternative reference regions, as well as the evaluation of side-separated nigral metabolism, did not overcome this issue (see Supplemental Table and Supplemental Fig. 2b). Therefore, the longitudinal evaluation of nigral metabolism with [^18^F]FDG PET may not accurately capture changes of neuronal viability in the substantia nigra along with PD progression^19–21^. Yet, an association with clinical parameters of disease severity and with striatal dopamine synthesis capacity was demonstrated in a rather selected cohort of PD patients without cognitive impairment^8^. Whereas these findings of impaired nigral metabolism in PD were acquired with a dedicated, high-resolution brain PET system^8^, the present study shows that recently introduced clinical whole-body PET/CT systems with fully digital detector technology also offers an improved, sufficient image quality and spatial resolution^22^ allowing for a reliable assessment of the substantia nigra. Still, further studies are needed to define optimal techniques.

This study has some limitations. First, the quantification of tracer uptake in small brainstem nuclei is susceptible for partial volume effects. We did not account for this, e.g. by using morphologic imaging for partial volume correction^23^. Instead, we chose a standardized template-based approach for volume delineation and quantification that does not require additional imaging and data processing but is easy to be implemented in clinical routine and to be reproduced. The comparison of PET- and magnetic resonance imaging (MRI)-based stereotactical normalization in those patients and controls with available T1 MPRAGE sequence supports the validity of the current PET-based approach (see Supplemental Table and Supplemental Fig. 2a). Second, the clinical diagnosis of NP as reference standard was not blinded towards clinical PET assessment and may bias disease groups towards patients with typical PET findings. However, substantia nigra metabolism was not contemplated in the original diagnosis and was not part of clinical routine reads and reports. Thus, it is highly unlikely that this biased the present study focusing on the substantia nigra. Instead, we strongly believe that including [^18^F]FDG PET improved the validity of the reference diagnosis^5^ since the accuracy of the clinical diagnosis alone is around 80% only^24–27^. The analysis of a subgroup of patients with available [^123^I]FP-CIT SPECT shows comparable effect sizes and confirms that the overall findings of this study are robust (see Supplemental Fig. 1a).

In summary, the present study shows in a large and clinically well-characterized cohort that glucose metabolism of the substantia nigra can distinguish patients with NP from controls with good diagnostic accuracy and can be used as a marker of nigral degeneration. Its evaluation is particularly valuable in PD patients without elevated PDRP expression and may thus help to narrow the diagnostic gap of [^18^F]FDG PET in neurodegenerative parkinsonism (i.e. identification of patients with PD without cortical involvement).

## Methods

### Participants

We retrospectively screened the records of the Department of Nuclear Medicine, Medical Center—the University of Freiburg (from January 2018 until April 2021) for patients who had undergone brain [^18^F]FDG PET on a fully digital PET/CT system for the differential diagnosis of NP. Two movement disorder specialists (NS, MR) made a consensus diagnosis based on all available anamnestic, clinical, and diagnostic information. One hundred seventy one patients met the criteria of probable PD (*n* = 115), MSA (*n* = 35), or PSP (*n* = 21) and were therefore enrolled in this study^2–4^. We excluded patients with corticobasal degeneration because of the small sample size (*n* = 5). The consensus diagnosis was not blinded to the clinical reports of PET imaging (though not yet contemplating nigral metabolism; see Discussion) and integrated the results of [^123^I]FP-CIT SPECT in 75 patients (PD, *n* = 62; MSA, *n* = 7; PSP, *n* = 6). The latter consistently showed a pathologically reduced dopamine transporter (DAT) availability in all patients. Thirteen HC subjects (54% male, 68.4 ± 6.8 years) recruited by local advertisement combined with 35 control patients (51% male, 65.3 ± 9.0 years) derived from a prior study^28^ served as the control cohort. HC were healthy according to medical history (no neurologic or psychiatric condition or any other relevant comorbidity) and unimpaired on neuropsychological evaluation, had no neurological deficit on clinical examination and had normal MRI findings of the brain. For demographics and patient characteristics, see Table 1.

### Imaging

PET emission data were acquired 50 min after intravenous injection of 209 ± 12 MBq [^18^F]FDG for 10 min on a fully digital PET/CT system (Vereos, Philips Healthcare). Fully-corrected emission datasets were reconstructed with low-dose CT-based attenuation correction and the vendor-specific, line-of-response time-of-flight ordered subsets 3D iterative reconstruction algorithm employing spherically symmetric basis functions (BLOB-OS-TF reconstruction; number of iterations = 5, number of subsets = 11, 2 mm Gaussian post-filtering, resulting voxel size = 1.0 × 1.0 × 1.0 mm^3^). This yielded a reconstructed, isotropic image resolution of approximately 4.5–5 mm full width at half maximum. Of note, we did not employ resolution recovery to avoid Gibb's artifacts.

### Image processing and analysis

All processing steps were implemented with an in-house pipeline in MATLAB (The MathWorks, Inc., Natick, Massachusetts, United States) and Statistical Parametric Mapping (SPM12) software (www.fil.ion.ac.uk/spm).

### Stereotactical normalization

[^18^F]FDG PET scans were stereotactically normalized to an in-house [^18^F]FDG PET template in Montreal Neurologic Institute (MNI) space. For validation of PET-based stereotactical normalization, we additionally performed a MRI-based stereotactical normalization of [^18^F]FDG PET scans for those individuals who had an available T1 MPRAGE MRI sequence (*n* = 96 PD; *n* = 33 MSA; *n* = 21 PSP; *n* = 13 HC). For this, individual MRI scans were segmented and stereotactically normalized to MNI space using CAT12 software in SPM12. [^18^F]FDG PET scans were co-registered to MRI and then transformed into MNI space by applying an MRI-defined transformation matrix. Mean [^18^F]FDG uptake in SN from MRI-based stereotactical normalized data was correlated to the uptake derived from PET-based stereotactical normalized data by using Spearman’s r. Moreover, between-group differences were assessed. Absolute mean counts in the substantia nigra from PET- and MRI-based stereotactically normalized scans showed a strong correlation (*r* = 0.96, *p* < 0.001). Normalized mean FDG uptake in the substantia nigra derived from scans stereotactically normalized using MRI was significantly different among diagnostic groups and controls, supporting the results based on PET-based stereotactical normalization (Supplemental Table 1 and Supplemental Fig. 2a).

### Reference region

**Fig. 3:**
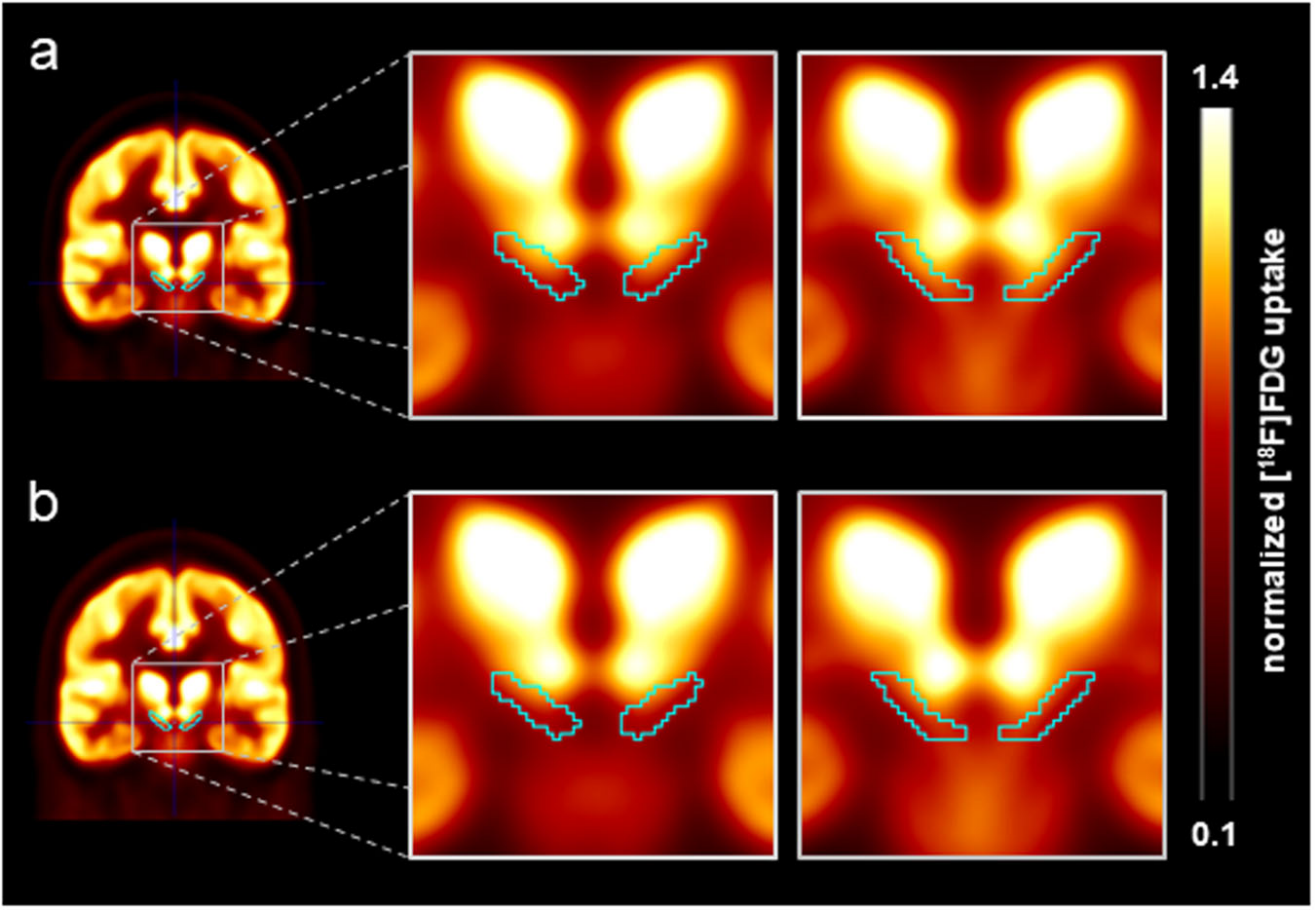
Average [^18^F]FDG PET images. [^18^F]FDG uptake of the substantia nigra normalized to the uptake in the composite reference region. Presented are coronal slices through the substantia nigra of the average scaled [^18^F]FDG PET image of the control group (**a**) and patients with PD (**b**). The teal contour delineates the volume of interest used for the substantia nigra.

To assess metabolism in the substantia nigra, we normalized the PET data to the uptake within a cohort-specific composite reference region (i.e., the mean individual’s uptake value in the substantia nigra was divided by the individual’s uptake value in the reference region). The cohort-specific reference region was defined by excluding the brainstem but including the substantia nigra, as well as regions with PD-, MSA-, and PSP-specific hypometabolism (top 20% of voxels with most pronounced hypometabolism within the MSA-, PSP- and PD-related patterns^9,29^) from a brain parenchyma mask (Supplemental Fig. 3). Anatomical definition of the substantia nigra was adopted from the Human Motor Thalamus atlas^30^ and binarized at the substantia nigra probability of >50% (Fig. 3). Mean normalized, side-averaged [^18^F]FDG uptake was calculated for each patient and control. To evaluate the effect of the reference region used for intensity scaling, we assessed the individual white matter (for all groups) and the pons (for PD and controls, but not for MSA and PSP due to midbrain atrophy in these diseases) as alternative reference regions. We observed significant differences in the mean bilateral [^18^F]FDG uptake in substantia nigra normalized to individual white matter uptake (ANCOVA, factor group: *p* < 0.001, *F* = 17.57, see Supplemental Table and Supplemental Fig. 2b) and to the pons (*p* < 0.001) confirming the results with the cohort-specific reference region.

### Side-separated nigral metabolism

When assessing [^18^F]FDG uptake for each side separately, we observed significantly different values among groups (ANCOVA, factor group: *p* < 0.001, *F* = 16.68, *p* < 0.001, *F* = 15.08, for left and right side, respectively). Mean [^18^F]FDG uptake of substantia nigra was significantly reduced in NP groups compared to controls (at least *p* < 0.01). Minimum [^18^F]FDG uptake of the both sides, showed similar significant results (ANCOVA, factor group: *p* < 0.001, *F* = 17.57; NP groups vs. controls all *p* < 0.01). For mean values of groups, please refer to Supplemental Table.

### Pattern analysis

For stratification of patients with PD, we assessed the expression of the well-defined PDRP using the ScAnVP software (SSM/PCA, The Feinstein Institute for Medical Research) in each patient with PD and HC. This pattern is characterized by relatively increased glucose metabolism of pallidothalamic, pontine and cerebellar clusters as well as decreased glucose metabolism in premotor and posterior parietal areas^9^. PD patients with a PDRP expression 2 standard deviations above the normal value of HC were considered to show an elevated PDRP expression (i.e., clear PD cases based on PET). In turn, those without an elevated PDRP expression constitute the clinically challenging subgroup of PD patients who are usually requested to undergo additional confirmatory imaging (e.g., DAT SPECT).

### Statistical analysis

Statistical analysis was performed with R software (version 4.1.0, http://www.R-project.org/). We compared demographic and clinical characteristics of patient and control groups with analysis of variance (ANOVA) followed by Tukey’s honest significance test. Between-group differences in normalized [^18^F]FDG uptake of the substantia nigra (NP and the subgroups [PD, MSA, and PSP] vs. controls) were assessed using age-adjusted analysis of covariance (ANCOVA) followed by Tukey’s honest significance test. We estimated the effect sizes for pairwise comparisons with Cohen’s d. Receiver operating characteristics (ROC) analysis was employed to assess the diagnostic performance of nigral [^18^F]FDG uptake for differentiation between NP and its subgroups and controls by means of the area under the ROC curve (AUC). Furthermore, we determined cut-off values for mean normalized [^18^F]FDG uptake of the substantia nigra with a requested minimum specificity of 90%, in line with the clinical need for a confirmatory test (reduced [^18^F]FDG uptake of the substantia nigra defined as a positive or pathological case). We also performed ROC analyses for PD subgroups with and without elevated expression of the PDRP. Furthermore, we assessed group differences between controls and PD patients with early (Hoehn & Yahr ≤2.5) and late (Hoehn & Yahr >2.5) disease stages and short (<5 years) and long (>5 years) disease duration. For PD patients, the relationship between mean normalized [^18^F]FDG uptake, [^123^I]FP-CIT specific binding potential (equal to binding ratio −1^31^) in the putamen ipsilateral to the clinically most affected side (i.e., the most suitable region to detect changes of striatal DAT density along with PD progression^32^), disease duration and Unified Parkinson’s Disease Rating Scale (UPDRS) motor part 3 (off medication) was assessed by using age-adjusted Pearson’s correlation coefficient. The significance threshold was set to Bonferroni-corrected *p* < 0.05.

### Ethical approval and patient consent

All patients and controls gave written informed consent to [^18^F]FDG PET imaging. The local institutional review board of the University Hospital Freiburg (22/20) approved the retrospective analysis.

## ^Supplementary information^

Supplemental material

## Data Availability

The data supporting the findings of this study are available upon reasonable request.
