# Supplementary material for: Nigral glucose metabolism as a diagnostic marker of neurodegenerative parkinsonian syndromes
Source: NPJ Parkinsons Dis. 2022 Sep 29;8:123. doi: 10.1038/s41531-022-00392-x (PMC9519554; doi:10.1038/s41531-022-00392-x)
Supplement: Supplementary file 1 — Supplemental material [file 41531_2022_392_MOESM1_ESM.pdf]

Nigral glucose metabolism as a diagnostic marker ofneurodegenerative parkinsonian syndromes

Nils Schröter, MD,[1] Ganna Blazhenets, PhD[2] Lars Frings, PhD,[2, 3] Wolfgang H. Jost, MD,[4] Cornelius Weiller, MD,[1] Michel Rijntjes, MD,[1] Philipp T. Meyer, MD, PhD, [2] Joachim Brumberg, MD[2]

[1]Department of Neurology, Medical Center – University of Freiburg, Faculty of Medicine, University of Freiburg, Germany

[2]Department of Nuclear Medicine, Medical Center – University of Freiburg, Faculty of Medicine, University of Freiburg, Freiburg, Germany

[3]Center for Geriatrics and Gerontology Freiburg, Medical Center - University of Freiburg, Faculty of Medicine, University of Freiburg, Freiburg, Germany

[4]Parkinson-Klinik Ortenau, Wolfach, Germany

SUPPLEMENTAL TABLE

Supplemental Table 1. Mean (SD) normalized [^{18}F]FDG uptake of the substantia nigra across diagnostic groups					
	**PD**	**MSA**	**PSP**	**NP**	**Controls**
**MRI-based stereotactical normalization**					
	n = 96	*n* = 33	*n* = 21	*n* = 150	*n* = 13[a]
Mean bilateral	0.83 (0.07)*	0.83 (0.06)*	0.80 (0.06)**	0.82 (0.07)**	0.88 (0.04)
**Normalized to white matter**					
	n = 115	*n* = 35	*n* = 21	*n* = 171	*n* = 48
Mean bilateral	0.93 (0.07)***	0.94 (0.08)***	0.88 (0.07)***	0.93 (0.07)***	1.00 (0.07)
**Normalized to pons**					
	n = 115				*n* = 48
Mean bilateral	0.92 (0.08)***	NA	NA	NA	1.02 (0.08)
**Normalized to cohort-specific reference region**					
	n = 115	*n* = 35	*n* = 21	*n* = 171	*n* = 48
Mean bilateral	0.80 (0.06)***	0.81 (0.08)***	0.77 (0.06)***	0.80 (0.07)***	0.87 (0.05)
Left	0.79 (0.06)***	0.80 (0.08)**	0.75 (0.07)***	0.78 (0.07)***	0.85 (0.05)
Right	0.82 (0.07)***	0.82 (0.08)***	0.78 (0.07)***	0.81 (0.07)***	0.88 (0.06)
Minimum of left and right	0.78 (0.07)***	0.78 (0.07)***	0.74 (0.07)***	0.78 (0.07)***	0.86 (0.05)

PD, Parkinson’s disease; MSA, multiple system atrophy; PSP, progressive supranuclear palsy; NP, neurodegenerative parkinsonian syndromes; n, number of subjects. SD, standard deviation. Differences in comparison to controls: * $p < 0.05$, ** $p < 0.01$, *** $p < 0.001$. [a] compared to healthy controls only

SUPPLEMENTAL FIGURES

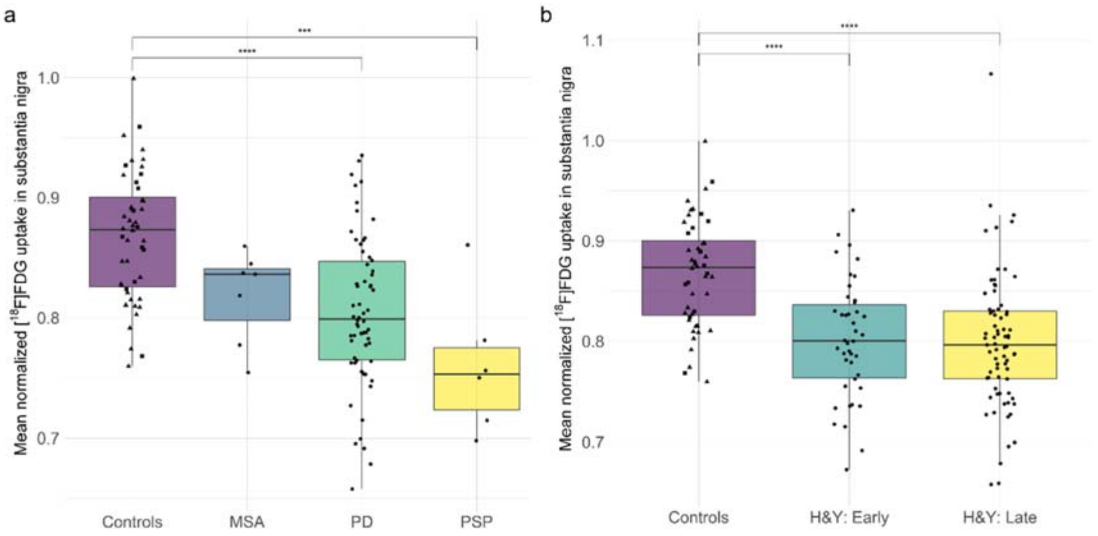

Supplemental Figure 1. Mean normalized [^{18}F]FDG uptake of the substantia nigra across diagnostic groups with [^{123}I]FP-CIT SPECT-confirmed diagnosis and controls (a) and in PD patients with early and late stages of the disease (b). Squares indicate healthy controls, triangles control patients, and dots represent individual patients' values. Center line, median; box limits, upper and lower quartiles; whiskers, 1.5x interquartile range; points, outliers. Significance threshold: **** p < 0.0001; *** p < 0.001. MSA, multiple system atrophy; PD, Parkinson's disease; PSP, progressive supranuclear palsy; H&Y, Hoehn & Yahr stage.

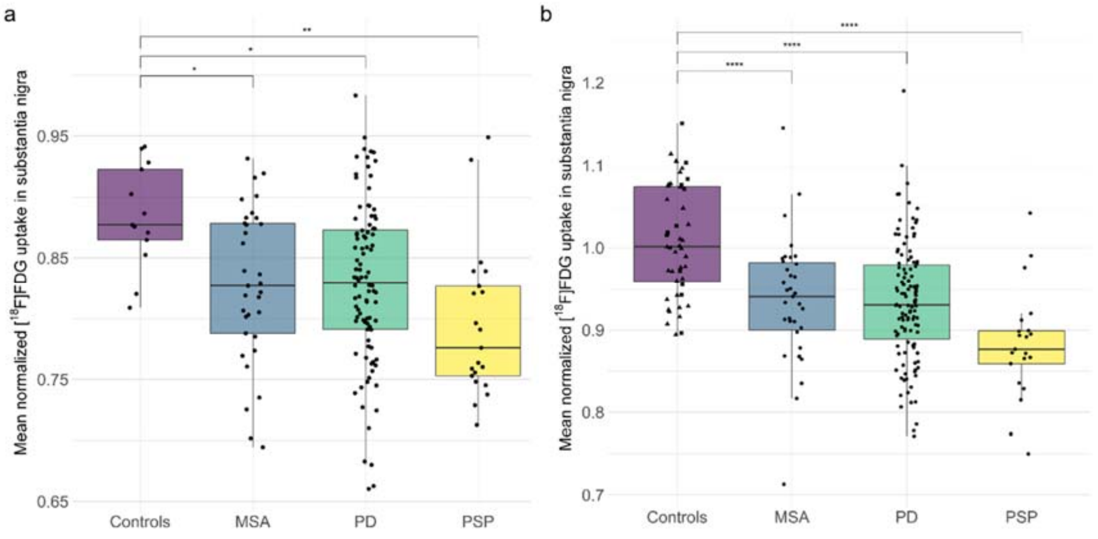

Supplemental Figure 2. Mean normalized [^{18}F]FDG uptake of the substantia nigra across diagnostic groups and controls. a, mean normalized [^{18}F]FDG uptake in the substantia nigra derived from PET scans stereotactically normalized using MRI-based transformation. MRI scans were available only for 13 healthy controls. Dots represent individual subjects' values. b, mean [^{18}F]FDG uptake of the substantia nigra across diagnostic groups and controls normalized to the individual uptake in white matter. Squares indicate healthy controls, triangles control patients, and dots represent individual patients' values. Center line, median; box limits, upper and lower quartiles; whiskers, 1.5x interquartile range; points, outliers. MSA, multiple system atrophy, PD, Parkinson's disease, PSP, progressive supranuclear palsy. Significance threshold: * p < 0.05; ** p < 0.01, **** p < 0.0001.

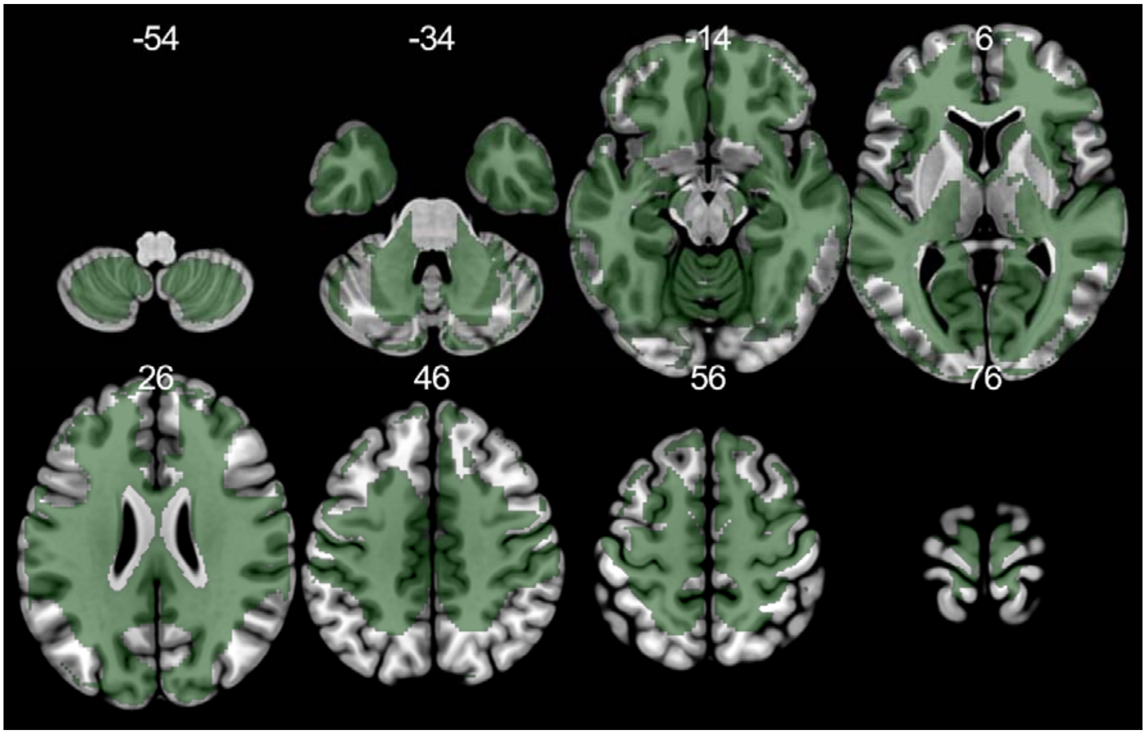

Supplemental Figure 3. Voxels included into the cohort-specific reference region for PET data normalization (green) overlaid on single-subject MRI template.
